# Learning from COVID-19: A Systematic Review of the IHR-SPAR Framework’s Role in the Pandemic Response

**DOI:** 10.3390/ijerph22050695

**Published:** 2025-04-27

**Authors:** Ida Santalucia, Michele Sorrentino, Claudio Fiorilla, Sabrina Tranquilli, Giordana Strazza, Paolo Montuori, Raffaele Palladino, Maria Fiore, Margherita Ferrante, Maria Triassi

**Affiliations:** 1 Department of Public Health, Experimental and Forensic Medicine, University of Pavia, 27100 Pavia, Italy; 2 Department of Public Health, University “Federico II” of Naples, 80131 Naples, Italy; 3 Department of Economics, Law, Cybersecurity, and Sports Sciences, Parthenope University, 80133 Naples, Italy; 4 Department of Political Science and Communication, University of Salerno, 84084 Fisciano, Italy; 5 Department of Primary Care and Public Health, School of Public Health, Imperial College, London SW7 2AZ, UK; 6 Interdepartmental Research Center in Healthcare Management and Innovation in Healthcare (CIRMIS), 80131 Naples, Italy; 7 Department of Medical Sciences, Surgical and Advanced Technologies “G.F. Ingrassia”, University of Catania, 95124 Catania, Italy; 8 International Society of Doctors for Environments—ISDE, Catania Section, 95100 Catania, Italy; 9 Research Center in Nanomedicine and Pharmaceutical Nanotechnology, Department of Drug and Health Sciences, University of Catania, 95124 Catania, Italy

**Keywords:** International Health Regulations (IHR), SPAR, COVID-19, systematic review

## Abstract

The International Health Regulations (IHR) provide a global framework for health security, requiring annual reporting on 35 indicators across 15 core capacities via the State Parties Annual Reporting (SPAR) tool. The COVID-19 pandemic exposed gaps in the IHR framework and monitoring systems, prompting calls for reform. This systematic review analyzed the correlations between IHR-SPAR scores and pandemic outcomes across nine studies (2020–2024), selected using the PRISMA guidelines. The study quality was assessed using the Joanna Briggs Institute’s tool for cross-sectional studies. Of 1019 screened studies, nine met the inclusion criteria. Higher SPAR scores generally correlated with lower COVID-19 incidence and mortality, although some high-scoring countries experienced severe outbreaks. Middle-income countries showed the greatest improvement, particularly in risk communication and emergency response, while zoonotic disease capacities saw little progress. While the SPAR tool aids monitoring, it requires revisions to better reflect real-world pandemic responses. High SPAR scores do not always indicate effective crisis management. This study recommends integrating more dynamic, operational, and context-sensitive indicators to enhance the global preparedness for future health emergencies.

## 1. Introduction

The International Health Regulations (IHR) are the cornerstone framework for global health security, established as a nearly universally recognized World Health Organization (WHO) treaty with 196 States Parties [1]. The primary purpose of the IHR is to safeguard global health by preventing, protecting against, and responding to international public health threats in a way that minimizes disruptions to international traffic and trade [2]. One of the IHR’s critical functions is the declaration of Public Health Emergencies of International Concern (PHEIC), which enables a coordinated global response to significant health risks [3].

Initially adopted by the World Health Assembly in 1969, the IHR have undergone several amendments in response to evolving global challenges, such as the expansion of international trade and travel. Following the 2002–2004 SARS outbreak, significant revisions were made in 2005 to bolster global preparedness and the response mechanisms. These updates, which came into force in 2007, created a legal framework for the management of public health events with the potential to spread internationally [4]. Notably, the IHR are the only international legal instrument that empowers the WHO to function as the main global surveillance system for health threats [5,6].

To ensure that signatory countries develop the necessary capabilities for the detection, assessment, and reporting of public health events, the IHR Monitoring and Evaluation Framework was established. This framework includes several tools—the State Parties Annual Report (SPAR), Joint External Evaluation (JEE), After Action Reviews (AAR), and Simulation Exercises (SimEx)—to assess weaknesses and gaps in national health systems [7].

Under Article 54 of the IHR, each State Party must annually report its progress in implementing the required capacities using the SPAR tool [1], which evaluates 35 indicators across 15 core capacities necessary to detect, assess, notify, report, and respond to public health risks and acute events. These core capacities are grouped into three overarching domains: prevention (e.g., legislation and financing, zoonotic events, food safety), detection (e.g., surveillance, laboratory systems, human resources), and response (e.g., emergency operations, risk communication, points of entry). Each capacity is scored on a scale from 1 (no capacity) to 5 (sustainable capacity) based on self-assessment by national authorities. The SPAR tool enables countries to monitor progress, identify capacity gaps, and prioritize areas for improvement in health emergency preparedness. The average score across all indicators is often referred to as the IHR score, which provides a useful comparative benchmark of global preparedness and supports the broader objectives of the International Health Regulations. Despite being self-reported and subject to potential bias, SPAR remains a central mechanism within global health governance, helping to guide national and international efforts toward stronger, more resilient, and more responsive health systems. The SPAR questionnaire is issued annually after the World Health Assembly, and States Parties use a multisectoral approach to gather information from all sectors involved in implementing the IHR core capacities [8].

The Coronavirus Disease 19 (COVID-19) pandemic exposed significant weaknesses in the IHR and their monitoring systems, leading to widespread demands for reform. It became evident that the existing tools for pandemic prevention and response were inadequate [9]. In response, both the IHR Review Committee and the Independent Oversight and Advisory Committee for the WHO Health Emergencies Programme have recommended changes, incorporating lessons from the pandemic to enhance global health preparedness for future outbreaks [10].

This systematic review aims to evaluate studies that have examined the performance of countries during the COVID-19 pandemic using the IHR score, which represents the average of the SPAR assessments. The objective is to identify correlations between the IHR score and pandemic outcomes, such as incidence and mortality, to determine the effectiveness of the SPAR assessment system. Through this critical analysis, it is intended to provide recommendations to improve the monitoring system and strengthen global health preparedness for future emergencies.

## 2. Materials and Methods

This systematic review focused on examining the existing literature on the performance of countries during the COVID-19 pandemic based on the IHR score. The review adhered to the Preferred Reporting Items for Systematic Reviews and Meta-Analyses (PRISMA) guidelines [11].

### 2.1. Eligibility Criteria

We included only original research published in English and conducted across global settings. The studies had to specifically address the correlation between the IHR score and COVID-19 outcomes, defined as the measurable impacts of COVID-19, including but not limited to incidence rates, mortality, case–fatality ratios, vaccination coverage, and variant spread. Regarding geographical scope, no restrictions were applied, meaning that studies from any country or region were eligible for inclusion. A detailed breakdown of the eligibility criteria can be found in Table 1.

### 2.2. Search Strategy

Our primary search was conducted in PubMed/MEDLINE, with additional searches in Embase, PsycINFO (EBSCOhost), the Health Technology Assessment Database, and Web of Science (Clarivate). The search strategy was designed to capture studies evaluating performance in managing the COVID-19 pandemic based on the IHR score and SPAR indicators. Key search terms included “SPAR” OR “IHR” OR “International Health Regulation*”) AND (“1 January 2020” [PDAT]:“1 April 2024” [PDAT]).

### 2.3. Data Extraction and Quality Assessment

Three independent reviewers (M.S., C.F., I.S.) screened the titles and abstracts of the extracted articles, identifying those that met the inclusion criteria using Rayyan Artificial Intelligence [12], a tool tailored for systematic review support. Rayyan streamlines the screening process by allowing reviewers to independently tag articles, apply inclusion and exclusion criteria, and perform blind screenings, which helps to reduce potential bias and enhances the accuracy. Any disagreements were resolved by consulting the senior reviewer (P.M.). Data extraction was conducted through a two-step process: (1) the full-text review of eligible studies to identify relevant variables and (2) the organization of extracted data into a structured Excel sheet according to the author, country/region, timeframe, study design, SPAR indicators, outcome variables, and key findings. The study quality was assessed using the Joanna Briggs Institute (JBI) critical appraisal checklist for cross-sectional studies [13]. The assessment tool for cross-sectional studies included eight specific questions, each answered with ‘Yes’, ‘No’, or ‘Not Applicable’. The checklist evaluated eight criteria: (1) the clear definition of sample inclusion criteria; (2) the detailed description of study subjects and settings; (3) the validity and reliability of exposure measurement; (4) the use of objective, standardized criteria to measure conditions; (5) the identification of confounding factors; (6) stated strategies for the management of confounders; (7) the validity and reliability of outcome measurement; and (8) the use of appropriate statistical analyses. Quality categories were assigned as follows: high quality (>75%), moderate quality (50–75%), and low quality (≤50%). Quality assessment was conducted independently by two reviewers (M.S. and C.F.).

## 3. Results

### 3.1. Study Selection

The main search identified 1019 studies and using Rayyan Artificial Intelligence [12]; 40 studies were identified through reference lists. Upon a review of their titles and abstracts, 989 studies were deemed irrelevant and excluded. Subsequently, 30 publications underwent a full-text review, resulting in the selection of nine studies meeting the inclusion criteria. Gray literature was not considered, as well as conference papers, dissertations, letters, and editorials.

The 21 studies were excluded for the following reasons: eight studies did not focus on SPAR, five were not specifically focused on COVID-19, seven provided general overviews of COVID-19 and the IHR, and one study was excluded due to the unavailability of the full text.

**Figure 1:**
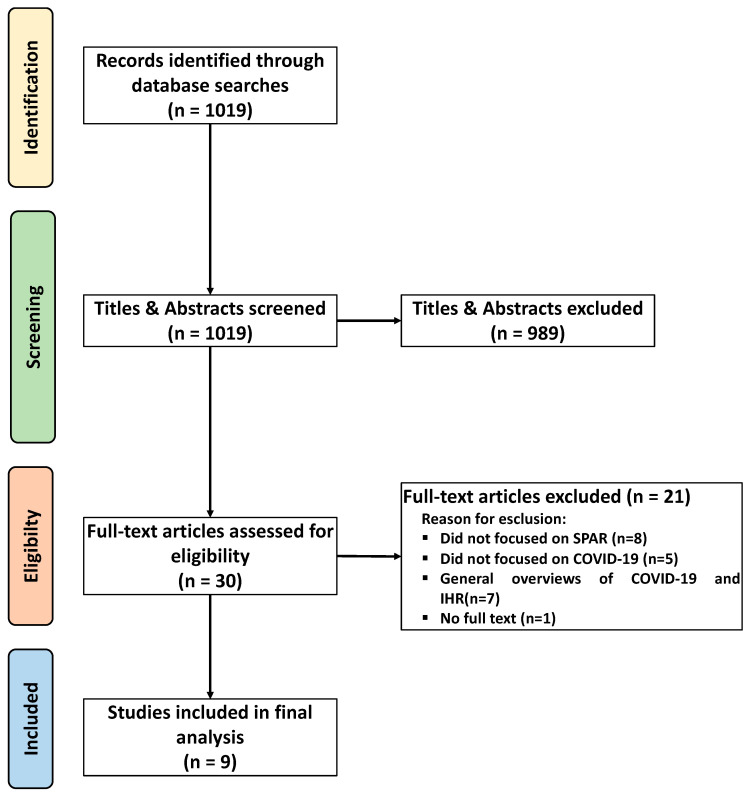
PRISMA flow diagram of literature search, abstract screening, and full article assessment for exclusion and inclusion criteria, with the most common reasons for exclusion detailed.

A visual representation of this selection process and the reasons for exclusion is provided in the PRISMA diagram (Figure 1).

### 3.2. Study Characteristics

The characteristics of the articles included are summarized in Table 2. Nine studies, spanning from 2020 to 2024, were selected for this review. All studies received high scores in the quality assessment. The publication years were as follows: one study in 2020 [14], two studies in 2021 [15,16], three in 2022 [17,18,19], two in 2023 [20,21], and one in 2024 [22].

Most of the studies were conducted in a global context, covering a diverse range of countries [15,16,17,18,19,21]. Additionally, one study focused on the South-East Asia region (SEAR) and Western Pacific region (WPR) [20]; another was a multi-country study encompassing Iran, Japan, South Korea, the United Kingdom, and the US [14]; and one was conducted in Lebanon [22].

The studies included in the review employed a variety of indicators to assess performance related to the COVID-19 pandemic. Specifically, COVID-19 mortality indices were utilized both individually [14,17,19] and in combination with incidence measures [15,18,20]. Additionally, one study integrated mortality and incidence with vaccination coverage [21], while another focused on analyzing variant replacement [16]. Lastly, one study did not utilize specific indicators but examined the SPAR framework during the COVID-19 pandemic [22].

### 3.3. Key Findings

The analyses show that higher SPAR scores were generally associated with lower COVID-19 incidence and a slower increase in case peaks, while the effective implementation of IHR capacities contributed to reducing the number of confirmed cases and deaths, although their effects diminished over time [15,18,21].

Moreover, high scores in specific areas—such as legislation, financing, coordination, food safety, human resources, health emergency frameworks, points of entry, and radiation emergencies—proved particularly significant for the trends in reported deaths and cases [18].

However, despite these overall trends, discrepancies persist, as evidenced by the fact that some countries with high SPAR scores experienced high cumulative mortality rates [14,19]. The analysis of score changes from 2019 to 2020 further revealed significant improvements among middle-income countries, while capacities related to zoonotic events and the human–animal interface remained largely unchanged [17]. Finally, examples such as Lebanon, which managed the pandemic more effectively than regional nations with lower IHR scores, underscore the crucial role that socioeconomic and regional contexts play in the overall management of the crisis [22].

## 4. Discussion

This study aimed to evaluate how countries performed during the COVID-19 pandemic based on the IHR score, particularly through the SPAR assessments. The study focused on identifying correlations between IHR scores and pandemic outcomes, such as incidence and mortality, to determine the effectiveness of the SPAR system in managing public health risks.

The systematic review highlighted several key findings that raise important questions about the effectiveness and reliability of the SPAR system in predicting and sustaining countries’ responsiveness to global health emergencies, particularly during the COVID-19 pandemic. Although the SPAR system was designed to monitor countries’ preparedness, the data collected show that the SPAR scores, while often high, did not always translate into effective pandemic management, raising questions about the current validity of the indicators used.

Countries with higher SPAR scores consistently exhibited lower COVID-19 incidence and mortality rates, along with a slower rise in peak incidence [14,15,18]. Additionally, there was an observed correlation between higher SPAR scores and reduced G* lineage replacement, emphasizing the importance of preparedness in curbing the spread of COVID-19 variants [16]. Similarly, a higher IHR score has been linked to more successful vaccination campaigns [21], further highlighting the importance of strong health systems, capable of efficiently implementing IHR core capacities, in mitigating early pandemic impacts. These findings align with broader evidence suggesting that better-prepared nations were able to report infections more promptly and vaccinate a larger proportion of their populations, significantly reducing the infection and mortality risks [23]. Preparedness has also been shown to reduce mortality, alleviate population stress, and promote public health and economic recovery following a pandemic crisis [24], further highlighting the critical role of strong health systems in fostering resilience beyond the SPAR assessments.

However, the COVID-19 pandemic exposed significant gaps in the current IHR framework, particularly in its ability to sustain long-term effectiveness. While the SPAR system proved useful during the early stages of the pandemic, helping countries to reduce their case numbers and mortality, its impact appeared to diminish as the pandemic progressed [21]. This raises concerns about the sustainability of the preventive measures embedded within the IHR framework, as countries struggled to maintain the same level of control over time. Although the IHR-SPAR has been credited with setting clear benchmarks to improve national capabilities in health security, it is limited by biases that may arise from country self-reporting [25,26]. These biases may contribute to discrepancies between the reported preparedness and actual pandemic outcomes, as seen during COVID-19. Notably, significant discrepancies were observed between countries with high SPAR scores, some of which still reported high cumulative death rates [14,19]. This reveals not only gaps in the IHR’s effectiveness but also regional disparities. For example, Lebanon, despite having relatively high IHR scores, managed the pandemic more effectively than the neighboring Syria and Jordan, demonstrating that, even within the same region, national responses to global health crises can vary significantly [22]. Such differences highlight that the SPAR scores alone may not fully capture the range of factors influencing a country’s pandemic management capabilities. Moreover, while the SPAR categories are interconnected and designed to reflect comprehensive health system preparedness, there is evidence that these categories are not directly linked to the actual capacity to manage pandemic outcomes [20]. This disconnection raises important questions about the SPAR system’s ability to accurately predict and improve a country’s response to global health threats. Therefore, while the IHR and the SPAR system have been crucial for early-stage pandemic preparedness, their limitations in sustaining long-term effectiveness and accounting for regional variations, compounded by potential self-reporting biases, suggest the need for further refinement.

Disparities in the SPAR score improvements between high-income and low- to middle-income countries became evident during the COVID-19 pandemic [17]. In 2020, many countries experienced a rise in SPAR scores, with middle-income nations leading this trend. Interestingly, most of the countries that did not see an improvement in their SPAR scores were high-income nations. Improvements were particularly notable in areas like risk communication, national health emergency frameworks, and points of entry. However, capacities related to zoonotic events and the human–animal interface have remained largely unchanged, underscoring the world’s ongoing vulnerability to future pandemics, particularly those of zoonotic origin. These events, which are the leading causes of PHEIC, pose significant future threats [27], especially in countries with weak health systems, insufficient preparedness, and inadequate surveillance mechanisms. Surprisingly, some high-income countries reported declines in key capacities, particularly in risk communication and national health emergency frameworks. Risk communication, which is vital in mitigating the impacts of pandemics like COVID-19, helps to create effective channels for the provision of timely and accurate information to the public [28,29,30]. The decrease in the SPAR scores among high-income nations may suggest that these countries used the pandemic as an opportunity to reassess and fine-tune their health system capabilities, aiming for more accurate and rigorous evaluations of their preparedness.

Conversely, the rise in the SPAR scores among low- and middle-income countries during the pandemic may indicate that these nations were underprepared before COVID-19, partly due to resource limitations and the need to prioritize other development-related expenditures [31]. However, the pandemic may have provided a critical moment for these countries to allocate more resources toward strengthening their capacities in infectious disease management, which likely contributed to their improved IHR scores during the first year of the pandemic. These improvements underscore the potential for growth in global health security, even in resource-constrained settings, when adequate focus and investment are directed toward preparedness measures.

In conclusion, while the SPAR system and IHR framework have played essential roles in pandemic preparedness, the COVID-19 crisis demonstrated their limitations, particularly in long-term effectiveness and addressing regional disparities. Future revisions should incorporate dynamic, real-time assessment tools and address self-reporting biases to ensure a more accurate reflection of global health security preparedness.

## 5. Policies

The findings of this review underscore the urgent need to revise the SPAR system and the broader IHR to address the lessons learned from the COVID-19 pandemic. The pandemic exposed critical weaknesses in the current regulatory framework, showing that it is insufficient in ensuring adequate preparedness and a timely response to public health emergencies. The revision of the SPAR indicators is necessary to incorporate dynamic factors such as governance, operational resilience, and the ability to rapidly mobilize resources [25]. While many governments are generally well equipped to detect outbreaks, they remain poorly prepared to prevent and respond to them.

One key recommendation is the inclusion of more flexible, simulation-based assessment tools like joint external evaluation (JEE), action after reviews (AARs), and simulation exercise (SimEx), which could help to identify and address operational gaps more effectively than static, self-assessed indicators of structural capabilities. Incorporating these dynamic tools into the IHR framework would enable countries to better respond to evolving public health threats and adjust their policies in real time.

Strengthening infection prevention and control measures is also crucial. Many factors contribute to the emergence and spread of infectious diseases, such as adherence to IPC protocols, climate-related pressures, population density, and national IHR capacities [32,33]. To mitigate the risks associated with these factors, countries must scale up evidence-based public health prevention strategies. These include promoting hand hygiene and respiratory etiquette, engaging local communities on the risks of outbreaks, and implementing effective public health response measures [32,34].

To improve pandemic management, decision-making processes must be both rational and feasible, with a clear focus on minimizing the impact of the crisis, particularly in reducing mortality and preventing healthcare system overload [35].

Ultimately, evidence-based policy planning and preparedness are critical in shaping the course of epidemics and reducing the infection risks and mortality rates, as demonstrated in previous outbreaks such as Ebola, meningitis, cholera, and influenza [36]. Strengthening health system preparedness is crucial to absorb the impact, respond effectively, and adapt to future public health emergencies [37].

## 6. Strengths and Limitations

The main strength of this study lies in its systematic approach to evaluating the performance of countries during the COVID-19 pandemic using the IHR-SPAR framework. By focusing on the correlation between SPAR scores and key pandemic outcomes, such as incidence and mortality, this review provides a comprehensive analysis of the effectiveness of the SPAR system. Additionally, the inclusion of a wide range of studies from different global settings enhances the generalizability of the findings. The study also emphasizes important considerations for improvements to the SPAR system, highlighting areas such as governance, operational resilience, and resource mobilization.

However, this study has several limitations. Firstly, the reliance on SPAR scores, which are self-reported by countries, introduces potential biases that could affect the accuracy of the assessments. This self-reporting may not accurately reflect the true preparedness of nations, as countries may overestimate their capabilities. Additionally, the SPAR scores considered were from different years across various studies, which could have introduced inconsistencies. Another limitation is the variability in the timing and contexts of the studies included, which may lead to discrepancies when comparing the results across different countries. Moreover, while our review included global and regional studies (e.g., SEAR/WPR and Lebanon), the scarcity of research from Africa, Latin America, and parts of Asia highlights a critical gap in the literature. This limits our ability to fully assess regional disparities and contextual challenges in pandemic preparedness. Future studies should prioritize these underrepresented regions to strengthen global health equity and ensure the IHR-SPAR framework’s applicability across diverse settings. Furthermore, in some studies, the COVID-19 mortality rates were not up to date, particularly in African countries, potentially affecting the results. The exclusion of gray literature and non-English studies may have introduced publication bias, as positive or neutral findings from low-resource settings or non-English-speaking regions might be underrepresented. Additionally, the reliance on self-reported SPAR scores, which are subject to overestimation, could have skewed the perceived preparedness of nations. These factors limit the generalizability of the findings and highlight the need for more transparent, standardized data reporting in future assessments. Lastly, while the systematic review provided critical insights into systemic gaps and contextual factors, its reliance on aggregated findings from published studies means that it may not reflect real-time or granular data dynamics. Future phases of our research program will address these limitations through direct analyses of raw SPAR and COVID-19 datasets, enabling the quantitative validation of correlations and the deeper exploration of regional disparities. This phased approach will ensure that our work builds iteratively, combining qualitative synthesis with empirical rigor to advance global health security policy. Despite these limitations, the findings contribute valuable insights into the strengths and gaps of the IHR-SPAR framework, offering a basis for future improvements in global health preparedness.

## 7. Conclusions

This systematic review underscores the limitations of the SPAR system as a monitoring tool, highlighting the need for substantial enhancements to better reflect the multifaceted nature of pandemic responses. High SPAR scores do not inherently equate to effective crisis management, revealing gaps in the current assessment framework. To strengthen global preparedness, future revisions should integrate a more comprehensive set of indicators that assess not only structural readiness but also the real-time operational capacity, adaptive response mechanisms, and socio-political dynamics. The COVID-19 pandemic serves as a pivotal moment to reform the IHR framework, ensuring a more resilient and responsive global health security system.

## Figures and Tables

**Table 1:** Eligibility criteria.

Eligibility Criteria
(P) Population
No restrictions
(I) Intervention
Assessment of national capacities using SPAR criteria
(O) Outcome
Correlation between SPAR scores and pandemic outcomes
Geographical Area (S)
No restrictions
Timeframe (T)
From 1 January 2020 to 1 April 2024
Other criteria
Written in English
Original research

**Table 2:** Study characteristics.

First Author, Publication Year [Ref.]	Country	Time Period	Study Design	Indicators	Outcome Variables	Key Findings	Quality Assessment
Wang et al., 2020 [14]	Iran, Japan, South Korea, UK, US	Up to September 2020	Cross-sectional	2018 SPAR for Iran; 2019 SPAR for Japan, South Korea, UK, and US	Cumulative number of deaths	Japan and South Korea in lower right of graph due to high SPAR and low cumulative deaths. US in middle with large population and deaths. Bubble sizes in similar areas showed low consistency; deaths and SPAR scores not clearly correlated.	>75%
Wong et al., 2021 [15]	Global (114 countries)	22 Jan to 2 Mar 2020 (start); 20 Feb to 31 Mar 2020 (end)	Cross-sectional	e-SPAR score	COVID-19 incidence/mortality	Higher e-SPAR scores linked to lower incidence per 100,000 within 30 days of first COVID-19 case. Similar trend for maximum incidence increase rate per 100,000. All models showed e-SPAR association with incidence/mortality. Thirteen IHR capacities linked to lower incidence/mortality.	>75%
Chen et al., 2021 [16]	Global	Start of pandemic to July 2020	Cross-sectional	2019 e-SPAR score	Variant replacement	Higher IHR scores (β −0.001, 95%CI −0.016, −0.001; *p* 0.034) correlated with lower lineage G* replacement levels.	>75%
Satria et al., 2022 [17]	Global (154 countries)	COVID-19 CFR data up to 31 March 2021	Cross-sectional	e-SPAR score	COVID-19 case fatality rate (CFR)	2020 e-SPAR scores different from 2019; 63.63% of countries saw increases, especially middle-income ones. Key gains in risk communication, national health emergency framework, and ports of entry, with zoonotic events unchanged.	>75%
Duong et al., 2022 [18]	Global (195 countries)	22 Jan 2020 to 17 June 2020	Cross-sectional	2019 or 2018 for IHR-SPAR	COVID-19 cases and deaths	Higher IHR-SPAR scores linked to lower COVID-19 cases and deaths per million. Key capacities associated with lower cases and deaths included legislation, coordination, food safety, human resources, etc.	>75%
Kachali et al., 2022 [19]	Global	First COVID-19 death to 60 days later, latest end of May 2020	Cross-sectional	IHR country rank	Cumulative reported deaths per million in first 60 days	Better-ranked countries showed higher mortality rates than lower-ranked ones.	>75%
Saengtabtim et al., 2023 [20]	South-East Asia (SEAR) and Western Pacific (WPR)	-	Cross-sectional	SPAR index	COVID-19 infections and deaths per million	SPAR capacities inter-related but no direct link to effective COVID-19 management outcomes.	>75%
Yuan et al., 2023 [21]	Global	From first confirmed COVID-19 case	Cross-sectional	IHR core index	COVID-19 confirmed cases, deaths, and vaccination coverage	Significant negative correlation between IHR capacity and COVID-19 cases/deaths over time. Higher IHR scores linked to fewer cases/deaths, leading vaccination efforts; gap narrowed over time.	>75%
Hassan et al., 2024 [22]	Lebanon	2020	Cross-sectional	SPAR index	-	Lebanon scored 4/5 (≤ 80%) in prevention, detection, response, etc., indicating capability for event management. Scored higher than Syria, Jordan.	>75%

## Data Availability

No new data were created or analyzed in this study. Data sharing is not applicable to this article.

## Abbreviations

The following abbreviations are used in this manuscript:

IHR	International Health Regulations
WHO	World Health Organization
PHC	Public Health Emergencies of International Concern
SPAR	State Parties Annual Report
JEE	Joint External Evaluation
AAR	After Action Reviews
COVID-19	Coronavirus Disease 19
PRISMA	Preferred Reporting Items for Systematic Reviews and Meta-Analyses
JBI	Joanna Briggs Institute
SEAR	South-East Asia
WPR	Western Pacific Region
CFR	Case Fatality Rate
SimEX	Simulation Exercise
